# Efficacy and toxicity of photon, proton, and carbon ion radiotherapy in the treatment of intracranial solitary fibrous tumor/hemangiopericytoma

**DOI:** 10.1186/s13014-024-02434-5

**Published:** 2024-03-29

**Authors:** Mike Ton, Maximilian Deng, Eva Meixner, Tanja Eichkorn, Anna Krämer, Katharina Seidensaal, Juliane Hörner-Rieber, Jonathan Lischalk, Klaus Herfarth, Jürgen Debus, Laila König

**Affiliations:** 1 Department of Radiation Oncology, University Hospital Heidelberg, Im Neuenheimer Feld 400, 69120 Heidelberg, Germany; 2 Present Address: Department of Radiotherapy, University Hospital Essen, Hufelandstraße 55, 45147 Essen, Germany; 3 Heidelberg Institute of Radiation Oncology (HIRO), Im Neuenheimer Feld 400, 69120 Heidelberg, Germany; 4 National Center for Tumor Diseases (NCT), Im Neuenheimer Feld 460, 69120 Heidelberg, Germany; 5 Clinical Cooperation Unit Radiation Oncology, German Cancer Research Center (DKFZ), Im Neuenheimer Feld 280, 69120 Heidelberg, Germany

**Keywords:** Intracranial solitary fibrous tumor, Intracranial hemangiopericytoma, Central nervous system solitary fibrous tumor, Central nervous system hemangiopericytoma, Postoperative radiotherapy, Proton radiotherapy, Carbon-ion radiotherapy, Photon radiotherapy

## Abstract

**Background:**

Solitary fibrous tumors (SFT) of the central nervous system are rare and treatment options are not well established. The aim of this study was to evaluate the clinical outcomes of radiotherapy (RT) and re-radiotherapy (re-RT) for de novo intracranial SFT and recurrent intracranial SFT.

**Methods:**

This retrospective study analyzed efficacy and toxicity of different RT modalities in patients who received radiotherapy (RT) for intracranial SFT at Heidelberg University Hospital between 2000 and 2020 following initial surgery after de novo diagnosis (“primary group”). We further analyzed the patients of this cohort who suffered from tumor recurrence and received re-RT at our institution (“re-irradiation (re-RT) group”). Median follow-up period was 54.0 months (0–282) in the primary group and 20.5 months (0–72) in the re-RT group. RT modalities included 3D-conformal RT (3D-CRT), intensity-modulated RT (IMRT), stereotactic radiosurgery (SRS), proton RT, and carbon-ion RT (C12-RT). Response rates were analyzed according to RECIST 1.1 criteria.

**Results:**

While the primary group consisted of 34 patients (f: 16; m:18), the re-RT group included 12 patients (f: 9; m: 3). Overall response rate (ORR) for the primary group was 38.3% (N = 11), with 32.4% (N = 11) complete remissions (CR) and 5.9% (N = 2) partial remissions (PR). Stable disease (SD) was confirmed in 5.9% (N = 2), while 41.2% (N = 14) experienced progressive disease (PD). 14% (N = 5) were lost to follow up. The re-RT group had 25.0% CR and 17.0% PR with 58.0% PD. The 1-, 3-, and 5-year progression-free survival rates were 100%, 96%, and 86%, respectively, in the primary group, and 81%, 14%, and 14%, respectively, in the re-RT group. Particle irradiation (N = 11) was associated with a lower likelihood of developing a recurrence in the primary setting than photon therapy (N = 18) (OR = 0.038; *p* = 0.002), as well as doses ≥ 60.0 Gy (N = 15) versus < 60.0 Gy (N = 14) (OR = 0.145; *p* = 0.027). Risk for tumor recurrence was higher for women than for men (OR = 8.07; *p* = 0.014) with men having a median PFS of 136.3 months, compared to women with 66.2 months.

**Conclusion:**

The data suggests RT as an effective treatment option for intracranial SFT, with high LPFS and PFS rates. Radiation doses ≥ 60 Gy could be associated with lower tumor recurrence. Particle therapy may be associated with a lower risk of recurrence in the primary setting, likely due to the feasibility of higher RT-dose application.

## Background

Solitary fibrous tumors (SFT) of the central nervous system (CNS) are fibroblastic neoplasms, which generally arise from the dura. While the true incidence and prevalence are difficult to ascertain due to its inconsistent nomenclature, previous series suggested that SFTs constitute < 1% of all CNS tumors[1]. Until the current update of the 2021 World Health Organization (WHO) classification of tumors of the CNS, SFTs were considered a tumor entity distinct from the formally known *hemangiopericytomas*.

Pathologist however found that histological distinction between those tumor entities proved to be very difficult in a lot of cases due to very similar histological and immunochemical features[2]. In the CNS WHO classification from 2016[3], it was acknowledged that both represent the same tumor entity[3], and in the 2021 CNS WHO classification, the name SFT was ultimately settled upon and the term hemangiopericytoma was omitted entirely[1].

Difficulty in diagnosis results from close radiographic features to meningioma, since both tumor entities share meningeal growth with contrast enhancement in both magnetic resonance imaging (MRI) and computer tomography (CT) images[2]. Tumor biopsy and pathology can provide definitive diagnosis. The current 2021 WHO classification delineates 3 distinct grades[1] and is oriented towards being a prognostic indicator, with higher grades displaying shorter overall and progression-free survival (OS, PFS)[3].

The rarity of SFTs results in an associated paucity of evidence regarding the most efficient treatment strategy and has made specific treatment recommendations challenging. Surgery seems to be the main domain of treatment of SFTs with most recent studies supporting gross tumor resection (GTR) as the main predicting factor for OS and PFS[4, 5]. Kim et al.[5] specifically report mean OS for patients with GTR to be 293.9 months compared to 151.4 months in patients with subtotal tumor resection (STR) (*p* = 0.012).

Common practices consist of surgery followed by postoperative radiotherapy (PORT), particularly in cases where only STR could be achieved, and macroscopic tumor tissue was left behind[4–8]. However, the efficacy of PORT remains topic of debate:

There is evidence for higher local control rates but no effect on OS[9], arguably because of the tumor’s propensity to metastasize [9–11], specifically Sung et al. note the 1-, 5-, 10- and 15-year recurrence rates with PORT of 0.0%, 0.0%, 55.5% and 70% and a 1-, 5- and 10 year recurrence rate without PORT of 7.7%, 55.1% and 73.1%, respectively.

Other studies support both longer PFS and OS with the addition of PORT [6, 12], Shiariti et al. found both a longer mean OS (254 vs 154 months; *p* = 0.2) and recurrence free interval (95 vs 70 months) of PORT vs no PORT.

Xiao et al. [13] on the other hand report no superior survival of patients having received surgery and PORT (both stereotactic radiosurgery (SRS) and intensity modulated radiotherapy (IMRT)) with local control rates of 58.6% in the surgery and PORT group vs 67.6% in the surgery alone group (*p* = 0.453).

As SFTs have a high tendency for recurrence[9], salvage therapies are also an active area of current research. Intracranial reirradiation (re-RT) carries high risks of toxicities and adverse events particularly when dose escalation is required[14, 15]. Fortunately, modern radiation techniques and modalities could provide broadened options for re-RT in patients with recurrent SFTs. Proton radiotherapy achieves highly precise energy deposition and superior sparing of surrounding tissues and organs at risk (OAR)[16]. Carbon ion radiotherapy (C12-RT) displays these characteristics in an even greater extent than protons, allowing greater protection of adjacent tissue and a higher relative biological effect (RBE) than photons or protons[17, 18].

In this retrospective patterns-of-care study, we aimed to explore the oncologic effectiveness and toxicity of radiation therapy for the treatment of SFT at Heidelberg University in the Department of Radiation Oncology. Consecutive cases were collected for PORT in both primary and salvage settings using different radiation modalities and ranges of radiation doses.

## Material and methods

### Patient population

We conducted a retrospective, single institution analysis of postinterventional (surgery or biopsy) radiotherapy of intracranial SFTs in the Department of Radiation Oncology at Heidelberg University Hospital. Radiotherapy was performed between June 2000 and October 2020. We included patients treated with both adjuvant radiotherapy following surgical resection or biopsy after initial diagnosis (primary group) and salvage RT following recurrence and repeat surgery (re-RT group). Ethics approval for the study was granted by the Heidelberg University ethics committee (#S-494/2021). Prior to treatment, every patient received a dedicated MRI scan of the tumor location, which was evaluated by a certified radiologist to assess macroscopic resection status. Pathology was performed with grading according to the then current CNS WHO classification at the time of diagnosis, spanning the CNS WHO classifications from 2000[19], 2007[20], 2016[3] to 2021[1].

### Treatment planning

Patients were immobilized with customized thermoplastic masks according to the irradiation technique used and treatment planning simulation CT scans with contrast agent with a slice thickness of 3 mm were obtained. Cranial magnetic resonance imaging of the brain (cMRI) was performed and fused with the planning CT-scan for target delineation. Gross tumor volume (GTV) included the macroscopic tumor and/or resection cavity. The clinical target volume (CTV) included the potential microscopic spread under consideration of all available information (surgical reports, pre- and postoperative imaging). Depending on the irradiation modality, an isotropic margin of 1–5 mm was added for the planning target volume (PTV) to account for geometric uncertainties and physical inaccuracies of the beam per physics recommendations.

### Treatment features

RT was performed with photons as intensity-modulated radiotherapy (IMRT) and 3D conformal RT (3DCRT) as well as stereotactic radiotherapy (SRS) via cyberknife. Particle therapy was applied as proton or carbon ion (C12) RT. For proton irradiation, an RBE of 1.1 was assumed according to the globally accepted clinical standard. For carbon ion re-irradiation, the biological dose was calculated using Local Effect Model I (LEM I) with an alpha/beta ratio of 2. In all cases, the irradiation and re-irradiation modality were based on both tumor board decision and institutional protocols. C12-RT was often preferred for re-irradiation because of its ability to better spare surrounding healthy tissue while achieving a higher radiobiological dose[17, 18].

### Outcome evaluation

Most patients received follow-up visits at Heidelberg University Hospital including a clinical examination as well as a contrast- enhanced MRI scan 6–8 weeks after completion of RT. This was repeated in 3- to 6-months intervals.

Local control (LC) was defined as no progressive disease of the treated primary, with treatment response being scored using the Response Evaluation Criteria in Solid Tumors (version 1.1). Treatment response was classified as complete response (CR), partial response (PR), stable disease (SD), or progressive disease (PD). OS was monitored from the first radiation fraction up to the last available follow-up or death. In case of reirradiation, OS was calculated from the beginning of the second radiation treatment. Progression free survival (PFS) similarly was calculated from the first radiation fraction until recurrence of the tumor, regardless of the recurrence being in-field or out-field, or death. Local progression free survival (LPFS) was defined from the start of irradiation until the first in-field recurrence. Treatment-related toxicity was evaluated and classified according to Common Terminology Criteria for Adverse Events Version 5.0 (CTCAE v5.0).

### Statistical analysis

Our statistical analysis aimed to assess the efficacy and pinpoint prognostic factors for radiation of SFTs. Kaplan–Meier method was carried out for survival numbers. Univariate cox proportional hazard models as well as odds-analysis were used to evaluate potential influence of patient, tumor, and treatment characteristics on survival times. Due to the small sample size and exploratory nature of the analysis multivariate analysis was not performed. A *p*-value ≤ 0.05 was defined as statistically significant and was calculated with fisher exact, cox regression and log-rank tests.

Statistical analysis was performed with IBM SPSS (Version 25.0, IBM, Armonk, NY, USA).

## Results

### Patient and treatment characteristics

Between June 2000 and October 2020, 34 patients (16 female, 18 male) received irradiation of an intracranial SFT in the Department of Radiation Oncology at Heidelberg University Hospital. Of these 34 patients, twelve (35.2%) received re-irradiation (re-RT) at our institution because of tumor recurrence. The median age at the time of radiation in the de novo situation was 47 years (20–71) and in the recurrence situation 59 years (39–75). The median follow-up period was 60 months (0–282) in the primary group and 26.5 months (0–72) in the re-RT group.

All patients had undergone surgical intervention as their first treatment prior to RT, with the majority performed as resection (76.5%, N = 26) and the remaining as biopsy alone (23.5%, N = 9). Salvage surgery was performed on 8 patients suffering from tumor recurrence (66.7%). For patients receiving initial treatment, median time between initial surgery and first RT was 4 months (1–105). For recurrent SFTs time between salvage surgery and re-RT was 7 months (2–28 months). On review of post-operative MRIs, the majority (58.8%, N = 20) of patients had residual macroscopic tumor before primary RT and the rest (41.2%; N = 14) no residual tumor.

In 29.4% of the cases (N = 10) tumors were classified WHO grade 2 and in 55.9% (N = 19) were WHO grade 3. The remainder of the cases (14.7%, N = 5) had unknown WHO grade.

Median size of PTV was 128 ml (21–383) in the primary group and 125.5 ml (32.0–210.0) in the re-RT group. RT doses adjusted by Equivalent Dose in 2Gy Fractions (EQD2) applied were between 47.9 and 75.0 Gy with the median being 60.0 Gy for primary RT and between 40.0 and 90.0 Gy with a median of 63.8 for re-RT. The high dose of 90.0 Gy is due to the SRS with cyberknife. The median single dose applied per fraction in the primary RT was 2.0 Gy (1.8–3.0) and in the re-RT was 3.0 Gy (2.0–18.0). In the primary group, 12 patients (35.3%) received 3D-CRT, 6 (17.6%) IMRT, 15 (44.1%) proton RT and 1 (2.9%) C12-RT. In the re-RT group, 1 patient (8.3%) was treated with 3D-CRT, 1 (8.3%) with IMRT, 2 (16.6%) with SRS and 8 (66.7%) with C12-RT. Detailed patient and treatment characteristics are displayed in Table 1.

**Table 1:** Patient characteristics

Total number of patients	34
Sex	
Male	18 (52.9%)
Female	16 (47.1%)
Age median in years (range)	
Primary situation	47 (20–71)
Re-RT situation	59 (39–75)
Number of RTs in primary situation	34
Number of RTs in recurrence situation (re-RT)	12
Time between initial diagnosis and radiation in months (range)	
Primary situation	9 (1–105)
Re-RT situation	138.5 (85–243)
Time between (re-)surgery and RT in months (range)	
Primary situation	4 (1–105)
Re-RT situation	7 (2–28)
Tumor localization	
Brain	34
Extent of primary operation as declared by surgeon in primary situation	
Resection	26 (76.5%)
Biopsy	8 (23.5%)
Residual tumor on planning MRI	
Primary situation	
Yes	20
No	14
Re-RT situation	
Yes	11
No	1
Radiation technique in primary situation	
3D-conformal RT	12 (35.3%)
IMRT/Tomo	6 (17.6%)
Stereotactic RT	0 (0%)
Proton RT	15 (44.1%)
C12-Ion RT	1 (2.9%)
Radiation technique for re-RT situation	
3D-conformal RT	1 (8.3%)
IMRT/Tomo	1 (8.3%)
Stereotactic RT	2 (16.6%)
Proton RT	0
C12-Ion RT	8 (66.7%)
Recurrence after first RT	
No	15 (44.1%)
Yes	14 (41.2%)
Unknown	5 (14.7%)
Median follow-up in months	
Primary situation	60 (0–282)
Re-RT situation	26.5 (0 – 72)
WHO grade	
WHO 2	10 (29.4%)
WHO 3	19 (55.9%)
Unknown	5 (14.7%)
Median PTV in ml (range)	
Primary situation	128 (21–383)
Re-RT situation	125.5 (6–254)
Median total RT Dose in Gy (range)	
Primary situation	60.0 (47.9–75.00)
Re-RT situation	63.8 (40.0–90.0)
Dose per fraction in Gy (range)	
Primary situation	2.0 (1.8–3.0)
Re-RT situation	3.0 (2.0–18.0)

The table shows the patient characteristics of the patient sample

RT = Radiation therapy, Tomo = Tomotherapy/helical IMRT, PTV = planning target volume, IMRT = Intensity modulated radiation therapy, ml = milliliters, Gy = Gray

### Oncological results

In the primary group, the overall response rate (ORR) was 38.3%, with 32.4% (N = 11) achieving CR and 5.9% (N = 2) showing PR. SD was confirmed in 5.9% (N = 2). 41.2% (N = 14) experienced PD, with 42.9% (N = 6) being in-field recurrences and 57.1% (N = 8) being out-field recurrences. The rest was lost to follow up (14.6%, N = 5) and did not receive follow-up examinations. The re-RT group showed a similar ORR with 36.4% (16.7% (N = 2) CR and 16.7% (N = 2) PR) but a higher rate of PD with 58.3% (N = 7) (57.1% (N = 4) in-field and 43.9% (N = 3) out-field recurrence) with one patient (8.3%) lost to follow up. The patients lost to follow-up were omitted from further response and survival analysis.

**Fig. 1:**
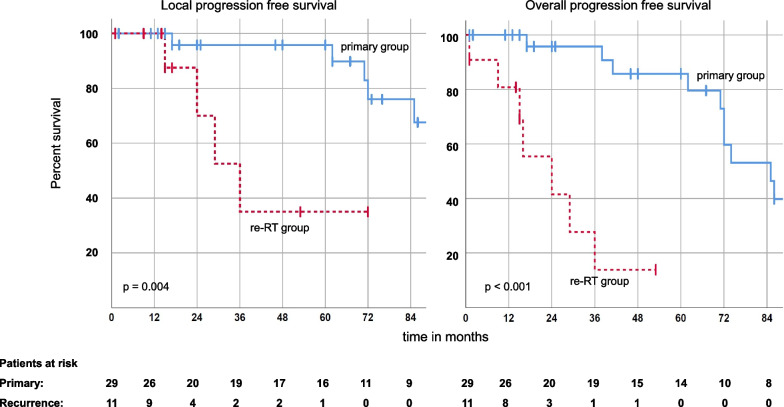
local and overall progression free survival. Kaplan Meier curves for local progression free survivals (LPFS) and overall progression free survival (PFS) for primary and re-RT group. Survival rates were significantly different between the groups (*p* = 0.004; *p* < 0.001)

1-, 3- and 5-year local progression free survival (LPFS) was 100.0%, 95.8% and 95.8% respectively for the primary group and was significantly longer (*p* = 0.004) than in the re-RT group, which showed a 1-, 3- and 5-year LPFS of 100%, 35.0% and 35.0% respectively. A similar pattern arose when considering overall PFS (including both in-field and out-field recurrences) with the primary group having a 1-, 3- and 5-year-PFS of 100%, 95.8% and 85.7%. The re-RT group showed a 1- and 3-year PFS of 80.8% and 13.9%, also with a significant (*p* < 0.001) difference regarding primary and re-RT situations (Fig. 1).

### Influences on oncological outcome

Particle RT (both proton and C12-ion radiotherapy, N = 11) proved to be significantly less likely to develop recurrences than photon RT (OR = 0.038; CI [0.004; 0.384]; *p* = 0.002). Survival times did not significantly differ (*p* = 0.750). No recurrence occurred in the single patient who received C12-RT. Detailed recurrence patterns of the primary situation are displayed in Table 2.

**Table 2:** Recurrence pattern analysis

	Recurrence			Total
	No	In-field	Out-field	
3D-conformal	2	5	5	12
IMRT/Tomo	3	1	2	6
Proton	9	0	1	10
C12-Ions	1	0	0	1
Total	15	6	8	29

Table shows recurrences of STFs in the de-novo group after radiotherapy (IMRT = intensity modulated radiation therapy, tomo = Tomotherapy/helical IMRT)

**Fig. 2:**
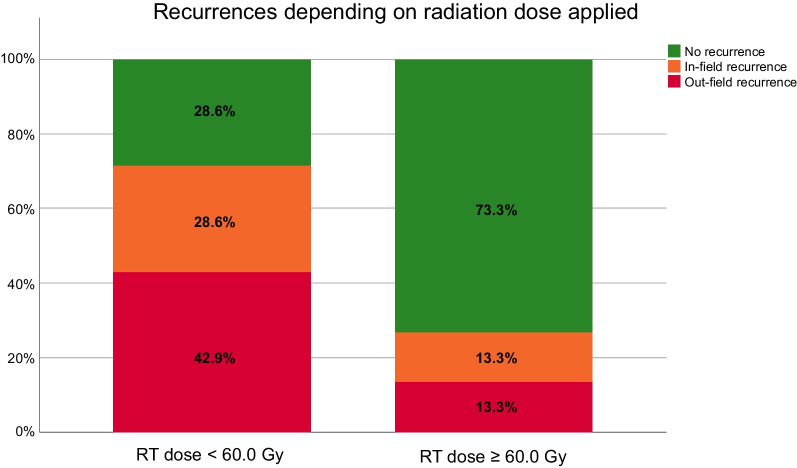
Recurrences depending on radiation dose applied. The figure shows a stacked bar count for recurrences after radiation with doses ≥ 60.0 Gy and < 60.0 Gy

Adjusting for modality, a significantly higher portion of patients reached a total radiation dose of 60.0 Gy with particle therapy than with photon radiation (*p* = 0.045). Median particle radiation dose was 60.0 Gy and for photon radiation 56.4 Gy. The median applied dose (adjusted by EQD2) was 60.0 Gy. The risk for both in- and out-field-recurrence was significantly lower for patients treated with ≥ 60.0 Gy in the primary RT (OR = 0.145; CI [0.029; 0.742]; p = 0.027). This was not significant when only looking at in-field-recurrences in the primary group (OR = 0.385; CI [0.058; 2.538]; *p* = 0.390). Survival times were not significantly different (*p* = 0.46) (Fig. 2).

When screening the pre-RT MRI images, we identified 65.5% (N = 19) macroscopic residual tumor vs 34.5% (N = 10) without macroscopic residual tumor (GTR vs. STR) and it did not show significant impact on the recurrence rate in the primary group (OR 0.485; *p* = 0.450). Log rank analysis on survival times did not show significance (*p* = 0.303) with median survival times for patients with macroscopic residual tumor on pre-RT MRI of 74.0 months and without macroscopic residual tumor of 91.0 months.

WHO grade approached significance with WHO grade 3 having an odds ratio of 4.67 for developing a recurrence compared to WHO grade 2 (*p* = 0.082), and an odds-ratio of 4.17 for developing an in-field recurrence (*p* = 0.061).

In the re-RT group, there was no significant difference in the likelihood of developing a recurrence (regardless of in-field or out-field) when comparing C12-RT vs photon-RT (OR = 1.66; CI [0.147; 18.874]; *p* = 1.0). Median PFS times showed a difference with 27.5 months for photon RT and 12.25 months for C12-RT (*p* = 0.717).

Females were overall significantly more likely to develop a recurrence (regardless of in-field or out-field) of SFTs compared to males (OR = 8.07; 95% CF [1.54, 42.32]; *p* = 0.014). Log rank analysis of survival times approaches significance (*p* = 0.08) with males having a median PFS of 136.3 months compared to 66.2 months for females. Mean survival times almost show no difference with 74.0 months for males and 71.0 months for females.

In Table 3 we ahve provided the detailed information for the pattern of recurrence.

**Table 3:** Analysis of recurrence patterns in the primary group

Patient (sex)	Age (years)	TTP (months)	RT dose (dose per fraction)	RT modality	Recurrence
Analysis of recurrence patterns in the primary group					
1 (f)	24	41	60.0 (2.0)	proton	out-field
2 (f)	65	72	59.4 (1.8)	3D-conformal	out-field
3 (f)	47	225	57.6 (1.8)	3D-conformal	out-field
4 (f)	44	86	57.6 (1.8)	3D-conformal	out-field
5 (f)	63	62	55.8 (1.8)	3D-conformal	in-field
6 (f)	36	85	60.0 (2.0)	3D-conformal	in-field
7 (f)	52	91	54.0 (1.8)	3D-conformal	in-field
8 (m)	52	72	60.0 (2.0)	IMRT	in-field
9 (f)	54	38	60.0 (2.0)	IMRT	out-field
10 (f)	64	17	59.4 (1.8)	3D-conformal	in-field
11 (f)	43	89	54.0 (1.8)	3D-conformal	out-field
12 (f)	68	71	54.0 (1.8)	3D-conformal	in-field
13 (m)	39	217	59.4 (1.8)	3D-conformal	out-field
14 (m)	29	74	54.0 (1.8)	IMRT	out-field

Patient (sex)	Age (years)	TTP (months)	RT dose (dose per fraction)	RT modality	Recurrence
Analysis of recurrence patterns in the recurrence group					
4 (f)	52	16	45.0 (3.0)	C12-ions	out-field
6 (f)	43	15	51.0 (3.0)	C12-ions	in-field
7 (f)	60	24	40.0 (2.0)	3D-conformal	in-field
8 (m)	59	36	51.0 (2.0)	C12-ions	in-field
9 (f)	59	9	51.0 (3.0)	C12-ions	out-field
10 (f)	65	1	51.0 (3.0)	C12-ions	out-field
13 (m)	57	29	18.0 (18.0)	Cyberknife	in-field

The table shows the detailed data on the patients who suffered a relapse. Listed are age at diagnosis, time to progression, radiation dose and modality, and recurrence-pattern

f = female, m = male, TTP = time to progress, RT = Radiotherapy, IMRT = Intensity modulated radiotherapy, C12-ions = Carbon ions

### Toxicity

In general, RT was well tolerated. Most adverse reactions were low grade I—II acute toxicities. In our patients, 31.4% reported no relevant acute toxicities, 48.6% had grade I toxicity (41.2% fatigue, 41.2% headache, 35.3% erythema, 29.4% localized hair loss, 23.5% others) and 20.0% grade II toxicity (28.6% erythema, 28.6% fatigue, 28.6% localized hair loss, 14.3% headache, 17.6% others). Detailed toxicity numbers are displayed in Table 4.

**Table 4:** Toxicities

	CTCAE I° (n = 17)	CTCAE II° (n = 7)
Fatigue	7	2
Headache	7	1
Erythema	6	2
Localized hair loss	5	2
Mucositis	1	1
Dysgeusia	1	0
Xerostomia	0	1
Otitis	1	0
Difficulty concentrating	1	0
Conjunctivitis	0	1

Number of mentioned adverse events. Only CTCAE I° and II° have been mentioned

CTCAE = Common Terminology Criteria for Adverse Events, n = number of patients affected

Patients could mention multiple adverse events

**Fig. 3:**
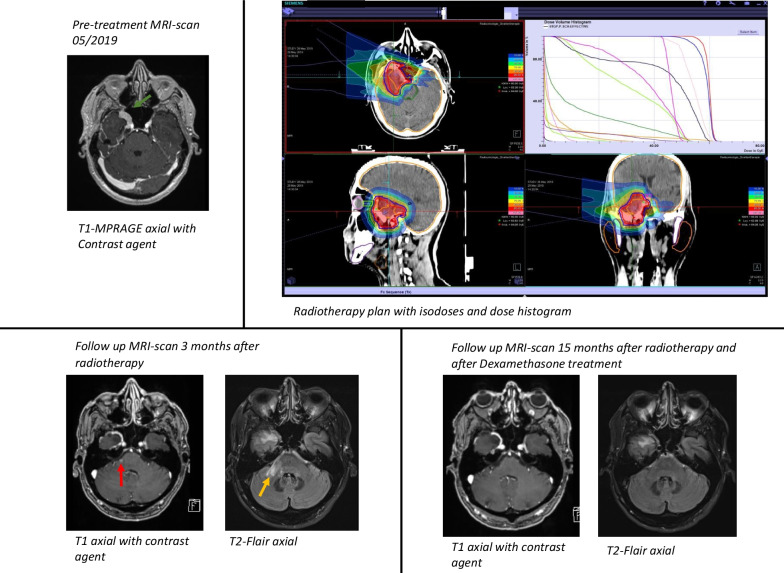
Case example. Images of patient A with a SFT of the sphenoid wing on the right side (green arrow). This patient underwent a biopsy and started proton radiotherapy 2 months after initial diagnosis. Response was excellent showing a CR and no recurrence during his 17 months of follow- up. Nevertheless, during follow-up, the patient suffered from RICE in the right pontine region (T1 contract enhanced (red arrow) with corresponding T2 FLAIR edema (yellow arrow), which resolved after dexamethasone therapy)

Nevertheless, 4 patients (11.4%) suffered from radiation induced contrast enhancement in the brain tissue (RICE) [14], all of which could be classified as CTCAE II°. Patient A had RICE after proton radiotherapy (60.0 Gy) in the primary situation. Treatment with Dexamethasone showed good response with decrease of RICE in the MRI. Patient B first had a 3D-CRT and a re-RT of a distant recurrence with tomotherapy and developed RICE thereafter. The cumulative dose adjusted by EQD2 was 114.7 Gy. Dexamethasone was only partially successful in reducing the size of RICE, but the patient showed no severe symptoms of RN. Patients C and D both had RICE after a local Re-RT with C12-Ions, patient C with a cumulative dose adjusted by EQD2 of 119.6 Gy and patient D of 123.8 Gy. Again, both patients were treated with Dexamethasone of which the former had good response and symptom alleviation, the latter did not pursue follow-up, therefore the outcome is unknown. We provide a detailed case example in Fig. 3.

## Discussion

Since SFTs of the CNS are extremely rare, there is very limited evidence regarding treatment strategies. As mentioned in the introduction, recent studies and meta analyses have shown favorable results for PORT[7–11]. This retrospective patterns-of-care study aimed to explore outcomes based on patient characteristics and treatment modality. To our knowledge, this analysis represents the largest cohort of patients having received RT with protons and C12 in the literature.

Nevertheless, due to the still limited number of patients and the retrospective nature of our study, definitive causal conclusions cannot be drawn. Consequently, the evidence found here can only give limited instructive guidance for clinical practice. Herein, we put our data in the context of existing literature on the disease in an attempt to deduct meaningful conclusions for the treatment of our patients.

Our survival results are mostly in line with earlier studies which already support radiotherapy as an effective adjunct treatment modality in combination with surgery upfront[6, 9–12]. Specifically Lee et al.[21] report significantly longer LC and OS rates in patients receiving PORT versus no PORT (5-year-LC-rates 97% vs 44%; HR 0.05 *p* = 0.002; and 10-year-OS-rates 83% vs 25%; HR 0.2 *p* = 0.008).

The OS rates with 96.0% 5 years after treatment of a primary SFTs are excellent and other studies report similar survival rates[10, 21, 22]. On the other hand, our analysis shows that outcome of recurrent SFTs is rather poor, with low survival times (14.0% after 3 years). Due to scarce data regarding salvage treatment of recurrent SFTs, the ideal treatment method is hitherto unclear. Our results demonstrate that salvage RT appears to be an option for retreatment, as acute toxicity seems to be tolerable without any toxicities preventing or delaying radiotherapy. Especially toxicity during re-RT was well tolerated in our patient cohort with only 48.6% reporting grade I toxicity (41.2% fatigue and headache, 35.3% erythema, etc.).

There is literature demonstrating cumulative RT doses of over 60.0 Gy to be favored to improve local control rates[25], with Ghia et al. stating superior local control with PORT with ≥ 60.0 Gy (HR 0.12 [0.01–0.95] *p* = 0.045)[25]. Our study underlines this finding with a lower incidence of local recurrences with RT doses ≥ 60.0 Gy, without showing significance (*p* = 0.390) which might be due to the limited patient numbers. Interestingly, when analyzing recurrence rate data generally, including both out-field and in-field recurrences, radiation doses ≥ 60.0 Gy had a significantly lower risk for a recurrence than radiation doses < 60.0 Gy (OR = 0.145; CI [0.029; 0.742]; *p* = 0.027). This, in turn, may reflect better local control and more efficient treatment of the primary tumor, as well as a lower likelihood of metastases and recurrence both within and outside the treatment field.

A central question to our study was the efficacy of different radiation modalities, specifically particle radiotherapy with carbon ions and protons. Studies focusing on these radiation modalities are very scarce and limited to case studies[26, 27].

Particle RT, especially in the primary state, seems to yield excellent response rates likely due to dose escalation feasibility. Only 1 of 10 patients treated with protons developed an out-field recurrence and the one patient treated with C12-Ions did not experience recurrence. Local control appears to be significantly superior to standard photon therapy. To our knowledge there is only one case report investigating radiotherapy with protons in a recurrence state of an orbital hemangiopericytoma [26]. The significant advantage of protons and C12-Ions compared to photons is the steeper dose gradient which allows higher dose applications both in the cases of proximity to organs at risk (i.e., chiasm, brain stem, optic tract, …) and the re-RT situation [17, 18]. This is reflected in our data, where doses of at least 60.0 Gy could be reached in a higher proportion of patients with particle RT compared to conventional photon treatment.

Similarly, there is only one case study in the recurrence situation of an extra cranial hemangiopericytoma treated with C12-RT [27]. In our study, most patients received C12-RT in the salvage setting with a 1-year LC rate of 68.6% and a median PFS of 12.0 months. Literature exploring the salvage setting mostly applies photon therapy; Sheen et al. [28] investigate SRS and report a 5 year LC rate of 76.0% and a median PFS of 21.0 months. The mean tumor volume in their study was 8.8 ml [28], whereas the mean GTV in our patients was 43.0 ml. This alone may point to a negative selection of our cohort and provide at least a possible explanation for our worse outcomes. Pretreatment of the patients was also not comparable to our study, with some patients having had received surgery only and SRS only after recurrence, some having had received SRS after initial definitive RT and some SRS after surgery and RT[28]. In our study all patients had received a surgical intervention and radiotherapy and only then re-RT with C12 or photons.

SRS is by definition only applicable in patients with small tumor volumes. C12-ions provide an option for re-RT for its higher RBE because of its higher linear energy transfer (LET) and – similar to protons – higher dose conformity with steeper dose gradients and therefore sparing of surrounding tissue[17, 27]. While our data shows a non-significant shorter median PFS of C12-RT vs photon RT in the salvage RTs of 12.25 vs 27.5 months we therefore still feel confident in at least further investigating ion radiotherapy especially in salvage situations to get a larger body of evidence for C12-RT.

As described in the backgrounds section there is general consensus that extent of resection (GTR vs STR) is a major contributor to survival times in SFT [4, 5]. This could not be confirmed in our study, both considering the extent of resection (EOR) as given by the surgeon as either resection or biopsy as well as our own assessment with pre-RT MRI imaging with either macroscopic tumor residual or none (GTR vs STR). Both did not show a significant correlation to tumor recurrence (*p* = 0.682; *p* = 0.450). A possible explanation could be our limited patient numbers. Regarding the pre-re-RT MRIs in the recurrence situation, we could identify that 11/12 patients had macroscopic tumor residuum/recurrence with 8 of them having had repeat surgery. This is not surprising since repeated surgery (especially after irradiation) is considerably more difficult and riskier to perform and has to be done more conservatively to avoid complications [29, 30]. There was no correlation between EOR in recurrent SFT regarding risk of tumor recurrence (*p* = 1.000). This could also be due to different tumor biology, already higher propensity for recurrence and the small patient sample size.

As with most high dose cerebral irradiations, there is a significant risk for radiation necrosis (RN) of brain tissue, especially after re-RT. Literature describes radiation induced contrast enhancement (RICE) as a term to morphologically describe said contrast enhancement in the brain tissue after RT, which can include blood–brain barrier lesions, pseudoprogression or even RN [14, 15]. Since only neurosurgery and pathology can safely distinguish between the different RICE lesions but is not always safely amenable in patients – especially after multiple surgeries already – definitive diagnosis and therefore treatment has proven to be difficult [14]. According to symptom severity antiedematous therapy with Dexamethasone is known to be alleviating of light symptoms [15] and RICE, with Bevacizumab (a VGEF-inhibitor) therapy offering a treatment option for RN [23, 24].

Over the course of re-RTs at our institution 4 patients developed RICE needing antiedematous treatment initiation. Dexamethasone as an initial treatment measure provided relief in 3 of 4 cases in our patients and symptoms didn’t prove to be severe. Bevacizumab therapy was not needed, and symptom alleviation and reduction of RICE could be achieved through Dexamethasone therapy only.

Our data suggest that women exhibit an overall higher risk of tumor recurrence than men. This finding is not yet understood. However, earlier surgical studies also show that female gender is associated with worse outcomes[22]. Potential biological or societal factors could predispose women for a worse outcome. Moving forward, we recommend that efforts should be undertaken to further examine gender differences in future studies.

Limitations to the current study include its retrospective character and the limited patient numbers as well as the heterogenous treatment techniques and applied doses. Of note, no new pathological evaluation had been performed as part of this retrospective review. This is a limitation of our study. In recent years new pathological findings have narrowed down and refined classification[1, 3]. We accepted the WHO grades without reclassification according to the then current WHO criteria. Our data supports the current consensus[8] and approaches significance in showing that WHO grade 3 SFTs have a higher risk for recurrence and especially in-field recurrences than WHO grade 2 tumors. The lack of significance is likely due to the limited patient numbers.

## Conclusion

Postoperative radiotherapy proves to be an efficient and safe treatment method with long survival times. We conducted the first retrospective patterns-of-care study including patients with proton and carbon ion radiotherapy. Our data shows a significantly superior outcome when applying doses ≥ 60 Gy with lower risk for recurrence in primary radiotherapy. Furthermore, particle radiotherapy showed significantly lower recurrence rates in this study, possibly due to the feasibility of higher dose delivery. Progression-free survival after salvage radiotherapy is significantly shorter than in the primary situation. C12 re-irradiation could be a feasible treatment modality, especially in larger tumor volumes. The lower survival rates may be due to the negatively selected patient cohort with large target volumes and should be further explored.

## Data Availability

The datasets generated and analyzed during the current study are not publicly available due to patient anonymity and their individual privacy but are available from the corresponding author on reasonable request.

## Abbreviations

3DCRT: 3D conformal radiotherapy
C12-RT: Carbon ion radiotherapy
cMRI: Cranial magnetic resonance imaging
CNS: Central nervous system
CR: Complete response
CT: Computer tomography
CTV: Clinical target volume
EOR: Extent of resection
EQD2: Equivalent Dose in 2Gy Fractions
GTR: Gross tumor resection
GTV: Gross tumor volume
Gy: Gray
IMRT: Intensity modulated radiotherapy
LEM I: Local effect model I
LET: Linear energy transfer
LPFS: Local progression free survival
OAR: Organ at risk
OR: Odds ratio
ORR: Overall response rate
OS: Overall survival
PD: Progressive disease
PFS: Progression free survival
PORT: Postoperative radiotherapy
PR: Partial response
PTV: Planning treatment volume
RBE: Relative biological effect
RICE: Radiotherapy induced contrast enhancement
RN: Radiation necrosis
RT: Radiotherapy
SD: Stable disease
SFT: Solitary Fibrous Tumor
SRS: Stereotactic radiosurgery
STR: Subtotal tumor resection
WHO: World Health Organization
